# The genome sequence of the Black Spongefly, *Sisyra nigra *(Retzius, 1783)

**DOI:** 10.12688/wellcomeopenres.20295.1

**Published:** 2023-11-09

**Authors:** Gavin R. Broad, Liam M. Crowley, James McCulloch, Natural History Museum Genome Acquisition Lab, University of Oxford and Wytham Woods Genome Acquisition Lab, Darwin Tree of Life Barcoding collective, Wellcome Sanger Institute Tree of Life programme, Wellcome Sanger Institute Scientific Operations: DNA Pipelines collective, Tree of Life Core Informatics collective, Darwin Tree of Life Consortium

**Affiliations:** 1 Natural History Museum, London, England, UK; 2 Department of Biology, University of Oxford, Oxford, England, UK

**Keywords:** Sisyra nigra, spongefly, genome sequence, chromosomal, Neuroptera

## Abstract

We present a genome assembly from an individual female *Sisyra nigra* (the Black Spongefly; Arthropoda; Insecta; Neuroptera; Sisyridae). The genome sequence is 372.6 megabases in span. Most of the assembly is scaffolded into 7 chromosomal pseudomolecules, including the X sex chromosome. The mitochondrial genome has also been assembled and is 16.34 kilobases in length.

## Species taxonomy

Eukaryota; Metazoa; Eumetazoa; Bilateria; Protostomia; Ecdysozoa; Panarthropoda; Arthropoda; Mandibulata; Pancrustacea; Hexapoda; Insecta; Dicondylia; Pterygota; Neoptera; Endopterygota; Neuropterida; Neuroptera; Hemerobiiformia; Sisyridae; *Sisyra*; *Sisyra nigra* (Retzius, 1783) (NCBI:txid279440).

## Background

*Sisyra nigra*, the Black Spongefly, or Spongillafly in North America, is a fascinating lacewing. The larvae eat freshwater sponges of the genera *Ephydatia* and *Spongilla* in lakes, ponds and slow-moving rivers ( Plant, 1997), apparently with little host specificity towards the sponges ( Poirrier, 1969). The female spongefly lays eggs on branches overhanging the water and the larvae are fully aquatic, at first free-swimming and then feeding within the sponge tissue, with many morphological and physiological adaptations to their unique niche ( Jandausch *et al.*, 2019). Pupation is on land. Adults disperse from water bodies and on overcast, humid nights can be light-trapped some distance from waterbodies, as was the case with these specimens collected for sequencing. Adults of *Sisyra nigra* are basically black, with an infuscate wing membrane, are small (about 5 mm long) and rest in a typical lacewing fashion, with the wings held in a tent-like shape over the body. They are mostly carnivorous, feeding on mites, insect eggs and sometimes aphids, as well as honeydew ( Devetak & Klokočovnik, 2016).

A Holarctic species ( Bowles, 2006), *Sisyra nigra* is one of only three British species of the family Sisyridae, all in the genus *Sisyra*, and is by the far the most widely distributed within Britain ( NBN Atlas Partnership, 2023; Plant, 1997). The combination of the entirely dark antennae and fore wings distinguishes *S. nigra* from the other two British species ( Plant, 1997). This is the first genome for a species of Sisyridae and as such will help towards piecing together the evolution and radiation of the Neuroptera, an ancient insect order with diverse life histories.

## Genome sequence report

**Figure 1.**
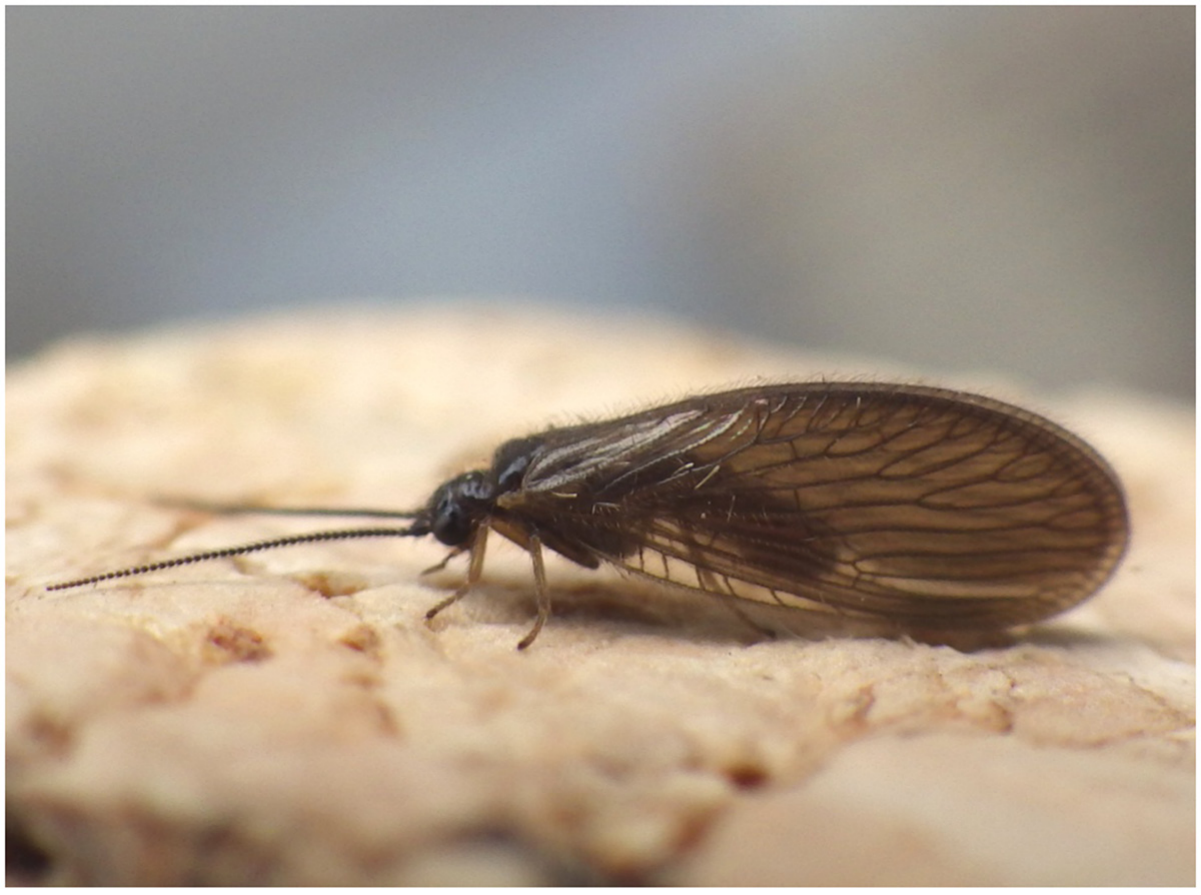
Photograph of the *Sisyra nigra* (inSisNigr1) specimen used for genome sequencing.

The genome was sequenced from one female *Sisyra nigra* ( Figure 1) collected from Kent, Tonbridge, Kent, UK (51.19, 0.29). A total of 55-fold coverage in Pacific Biosciences single-molecule HiFi long reads was generated. Primary assembly contigs were scaffolded with chromosome conformation Hi-C data. Manual assembly curation corrected 117 missing joins or mis-joins and removed 49 haplotypic duplications, reducing the assembly length by 2.16% and the scaffold number by 31.71%, and increasing the scaffold N50 by 0.43%.

**Figure 2.**
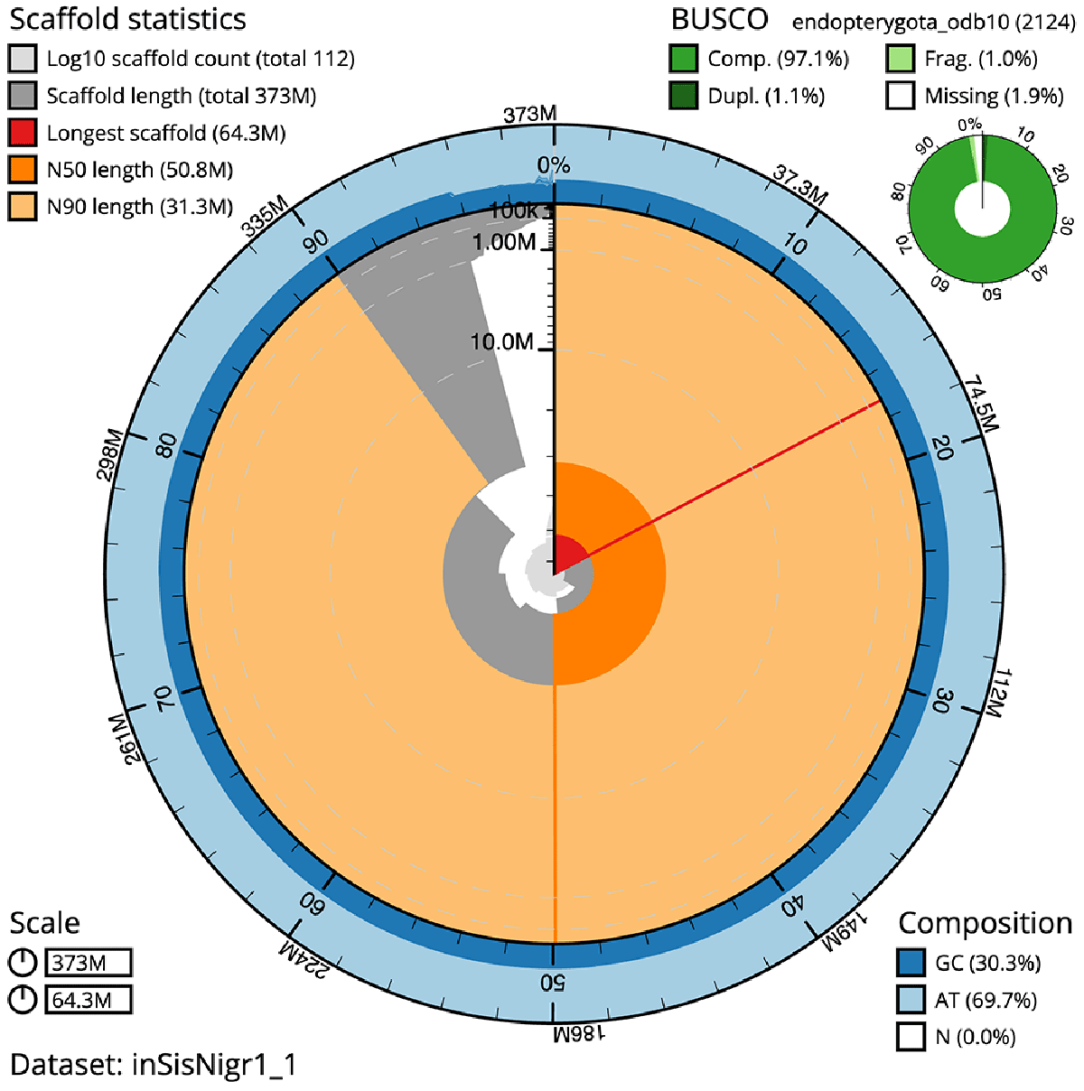
Genome assembly of *Sisyra nigra*, inSisNigr1.1: metrics. The BlobToolKit Snailplot shows N50 metrics and BUSCO gene completeness. The main plot is divided into 1,000 size-ordered bins around the circumference with each bin representing 0.1% of the 372,608,688 bp assembly. The distribution of scaffold lengths is shown in dark grey with the plot radius scaled to the longest scaffold present in the assembly (64,276,211 bp, shown in red). Orange and pale-orange arcs show the N50 and N90 scaffold lengths (50,790,609 and 31,345,168 bp), respectively. The pale grey spiral shows the cumulative scaffold count on a log scale with white scale lines showing successive orders of magnitude. The blue and pale-blue area around the outside of the plot shows the distribution of GC, AT and N percentages in the same bins as the inner plot. A summary of complete, fragmented, duplicated and missing BUSCO genes in the endopterygota_odb10 set is shown in the top right. An interactive version of this figure is available at https://blobtoolkit.genomehubs.org/view/Sisyra%20nigra/dataset/inSisNigr1_1/snail.

**Figure 3.**
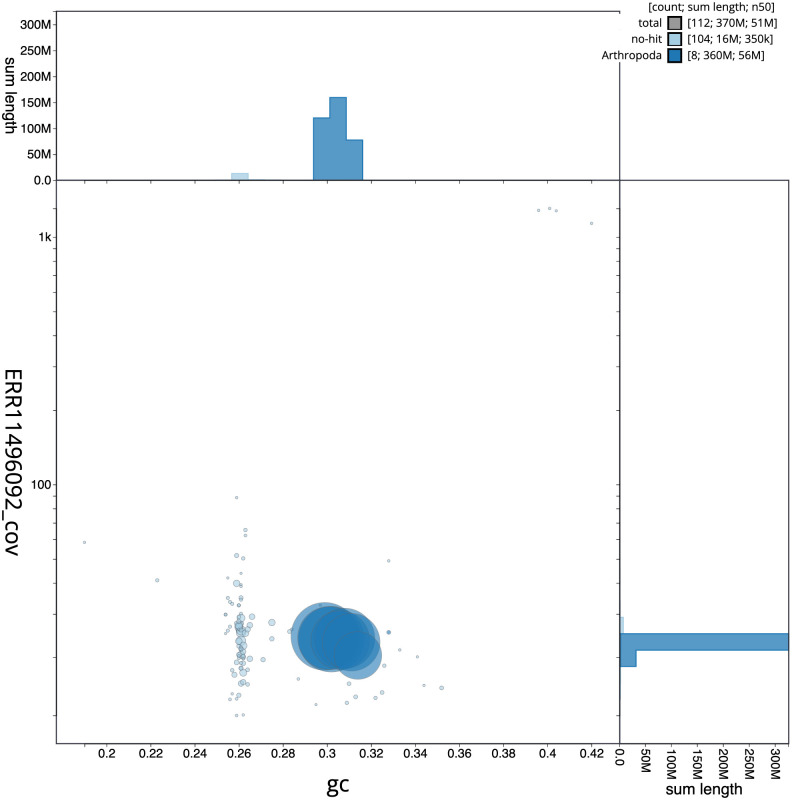
Genome assembly of *Sisyra nigra*, inSisNigr1.1: BlobToolKit GC-coverage plot. Scaffolds are coloured by phylum. Circles are sized in proportion to scaffold length. Histograms show the distribution of scaffold length sum along each axis. An interactive version of this figure is available at https://blobtoolkit.genomehubs.org/view/Sisyra%20nigra/dataset/inSisNigr1_1/blob.

**Figure 4.**
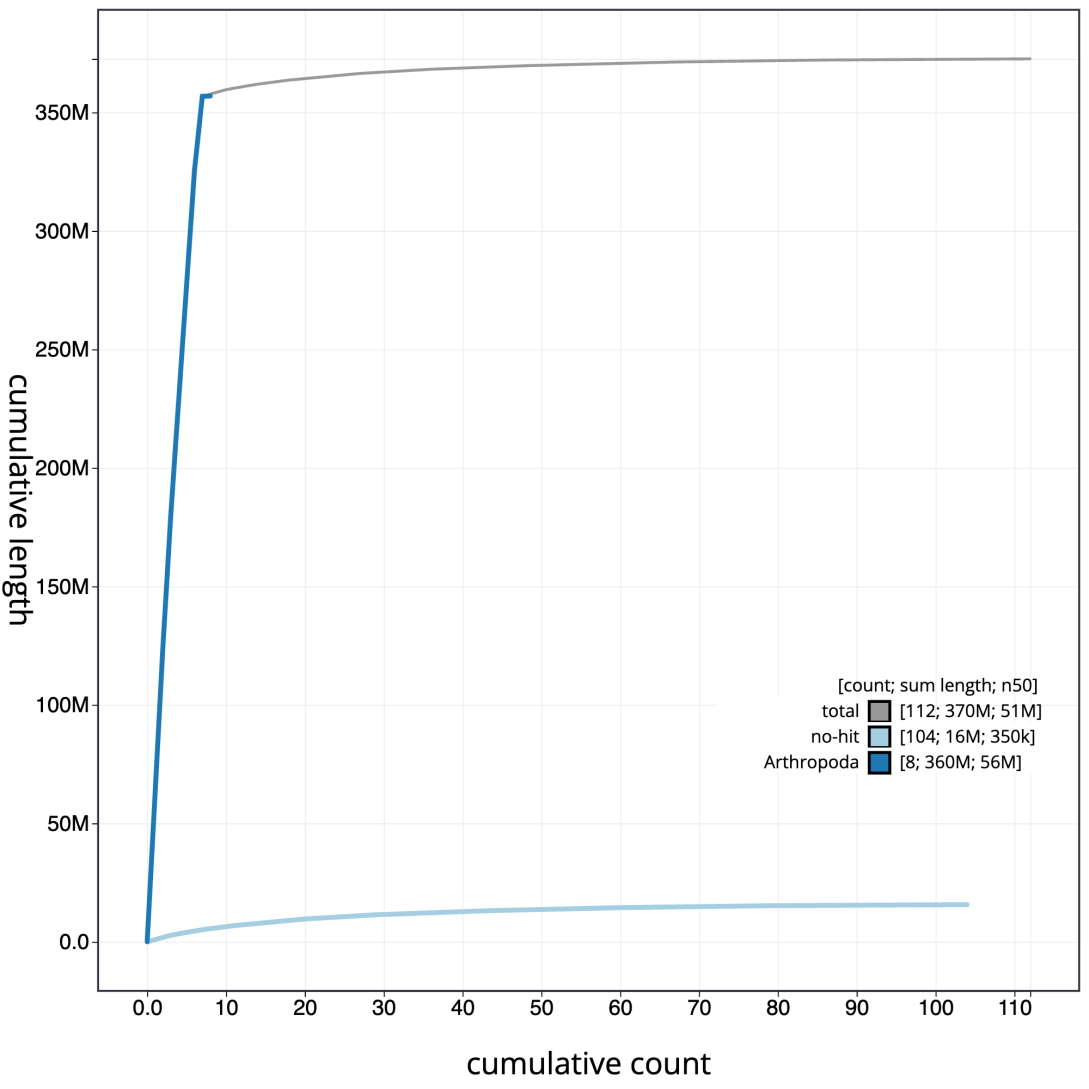
Genome assembly of *Sisyra nigra*, inSisNigr1.1: BlobToolKit cumulative sequence plot. The grey line shows cumulative length for all scaffolds. Coloured lines show cumulative lengths of scaffolds assigned to each phylum using the buscogenes taxrule. An interactive version of this figure is available at https://blobtoolkit.genomehubs.org/view/Sisyra%20nigra/dataset/inSisNigr1_1/cumulative.

**Figure 5.**
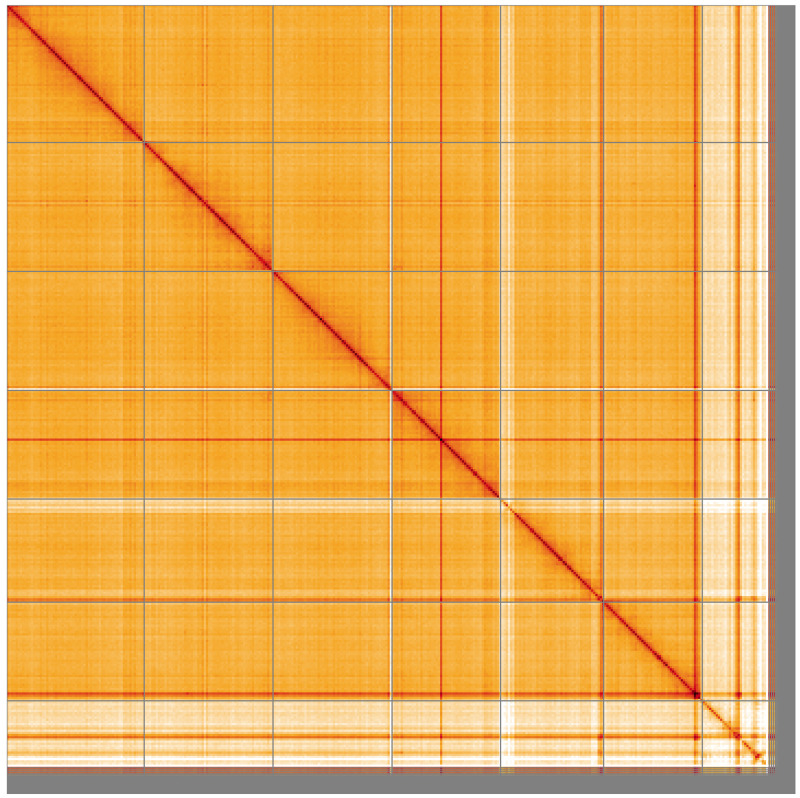
Genome assembly of *Sisyra nigra*, inSisNigr1.1: Hi-C contact map of the inSisNigr1.1 assembly, visualised using HiGlass. Chromosomes are shown in order of size from left to right and top to bottom. An interactive version of this figure may be viewed at https://genome-note-higlass.tol.sanger.ac.uk/l/?d=EWZtAmYIS12BnjH_wfvHGg.

The final assembly has a total length of 372.6 Mb in 111 sequence scaffolds with a scaffold N50 of 50.8 Mb ( Table 1). The snailplot in Figure 2 provides a summary of the assembly statistics, while the distribution of assembly scaffolds on GC proportion and coverage is shown in Figure 3. The cumulative assembly plot in Figure 4 shows curves for subsets of scaffolds assigned to different phyla. Most (95.76%) of the assembly sequence was assigned to 7 chromosomal-level scaffolds, representing 6 autosomes and the X sex chromosome. The X chromosome was identified based on reduced Hi-C signal (PacBio from female, Hi-C appears to be from male). An inversion between 20 Mb and 22 Mb on Chromosome 1 likely corresponds to difference between samples used for PacBio and Hi-C. Chromosome-scale scaffolds confirmed by the Hi-C data are named in order of size ( Figure 5; Table 2). While not fully phased, the assembly deposited is of one haplotype. Contigs corresponding to the second haplotype have also been deposited. The mitochondrial genome was also assembled and can be found as a contig within the multifasta file of the genome submission.

**Table 1.** Genome data for *Sisyra nigra*, inSisNigr1.1.

Project accession data		
Assembly identifier	inSisNigr1.1	
Assembly release date	2023-07-18	
Species	*Sisyra nigra*	
Specimen	inSisNigr1	
NCBI taxonomy ID	279440	
BioProject	PRJEB62618	
BioSample ID	SAMEA7521517	
Isolate information	inSisNigr1, female: whole organism (DNA sequencing) inSisNigr2, male (Hi-C data and RNA sequencing)	
Assembly metrics *		*Benchmark*
Consensus quality (QV)	60.3	*≥ 50*
*k*-mer completeness	100%	*≥ 95%*
BUSCO **	C:97.1%[S:96.0%,D:1.1%],F:1.0%,M:1.9%,n:2,124	*C ≥ 95%*
Percentage of assembly mapped to chromosomes	95.76%	*≥ 95%*
Sex chromosomes	X chromosome	*localised homologous pairs*
Organelles	Mitochondrial genome assembled	*complete single alleles*
Raw data accessions		
PacificBiosciences SEQUEL II	ERR11483521	
Hi-C Illumina	ERR11496094	
PolyA RNA-Seq Illumina	ERR12035194	
Genome assembly		
Assembly accession	GCA_958496155.1	
*Accession of alternate haplotype*	GCA_958496165.1	
Span (Mb)	372.6	
Number of contigs	353	
Contig N50 length (Mb)	3.1	
Number of scaffolds	111	
Scaffold N50 length (Mb)	50.8	
Longest scaffold (Mb)	64.3	

* Assembly metric benchmarks are adapted from column VGP-2020 of “Table 1: Proposed standards and metrics for defining genome assembly quality” from ( Rhie *et al.*, 2021). ** BUSCO scores based on the endopterygota_odb10 BUSCO set using v5.3.2. C = complete [S = single copy, D = duplicated], F = fragmented, M = missing, n = number of orthologues in comparison. A full set of BUSCO scores is available at https://blobtoolkit.genomehubs.org/view/Sisyra%20nigra/dataset/inSisNigr1_1/busco.

**Table 2.** Chromosomal pseudomolecules in the genome assembly of *Sisyra nigra*, inSisNigr1.

INSDC accession	Chromosome	Length (Mb)	GC%
OY292313.1	1	64.28	30.0
OY292314.1	2	60.28	30.0
OY292315.1	3	55.73	30.0
OY292316.1	4	50.79	31.0
OY292317.1	5	48.24	30.5
OY292318.1	6	46.14	31.0
OY292319.1	X	31.35	31.5
OY292320.1	MT	0.02	19.0

The estimated Quality Value (QV) of the final assembly is 60.3 with *k*-mer completeness of 100%, and the assembly has a BUSCO v5.3.2 completeness of 97.1% (single = 96.0%, duplicated = 1.1%), using the endopterygota_odb10 reference set ( *n* = 2,124).

Metadata for specimens, barcode results, spectra estimates, sequencing runs, contaminants and pre-curation assembly statistics are given at https://links.tol.sanger.ac.uk/species/279440.

## Methods

### Sample acquisition and nucleic acid extraction

A female *Sisyra nigra* (specimen ID NHMUK010634999, ToLID inSisNigr1) was collected in a light trap in Tonbridge, Kent, UK (latitude 51.19, longitude 0.29) on 2020-06-24. The specimen was collected and identified by Gavin Broad (Natural History Museum) and kept in DESS for 2 weeks before being frozen at –80°C.

The specimen used for Hi-C and RNA sequencing (specimen ID Ox002595, ToLID inSisNigr2) was collected using a sweep net in Wytham Woods, Oxfordshire (biological vice-county Berkshire), UK (latitude 51.77, longitude –1.34) on 2022-08-01. The specimen was collected by Liam Crowley and James McCulloch (University of Oxford) and identified by James McCulloch (University of Oxford).

High molecular weight (HMW) DNA was extracted at the Tree of Life laboratory, Wellcome Sanger Institute (WSI), following a sequence of core procedures: sample preparation; sample homogenisation; HMW DNA extraction; HMW DNA fragmentation; and fragmented DNA clean-up. The inSisNigr1 sample was weighed and dissected on dry ice (as per the protocol at https://dx.doi.org/10.17504/protocols.io.x54v9prmqg3e/v1). Tissue from the whole organism was disrupted using a Nippi Powermasher fitted with a BioMasher pestle ( https://dx.doi.org/10.17504/protocols.io.5qpvo3r19v4o/v1). HMW DNA was extracted by means of the Manual MagAttract protocol ( https://dx.doi.org/10.17504/protocols.io.6qpvr33novmk/v1). HMW DNA was sheared into an average fragment size of 12–20 kb in a Megaruptor 3 system with speed setting 30, following the HMW DNA Fragmentation: Diagenode Megaruptor®3 for PacBio HiFi protocol ( https://dx.doi.org/10.17504/protocols.io.8epv5x2zjg1b/v1). Sheared DNA was purified by solid-phase reversible immobilisation (SPRI) ( https://dx.doi.org/10.17504/protocols.io.kxygx3y1dg8j/v1). In brief, the method employs a 1.8X ratio of AMPure PB beads to sample to eliminate shorter fragments and concentrate the DNA. The concentration of the sheared and purified DNA was assessed using a Nanodrop spectrophotometer and Qubit Fluorometer and Qubit dsDNA High Sensitivity Assay kit. Fragment size distribution was evaluated by running the sample on the FemtoPulse system.

RNA was extracted from inSisNigr2 in the Tree of Life Laboratory at the WSI using the RNA Extraction: Automated MagMax™ *mir*Vana protocol ( https://dx.doi.org/10.17504/protocols.io.6qpvr36n3vmk/v1). The RNA concentration was assessed using a Nanodrop spectrophotometer and Qubit Fluorometer using the Qubit RNA Broad-Range (BR) Assay kit. Analysis of the integrity of the RNA was done using the Agilent RNA 6000 Pico Kit and Eukaryotic Total RNA assay.

All protocols developed by the Tree of Life laboratory are publicly available on protocols.io: https://dx.doi.org/10.17504/protocols.io.8epv5xxy6g1b/v1.

### Sequencing

Pacific Biosciences HiFi circular consensus DNA sequencing libraries were constructed according to the manufacturers’ instructions. Poly(A) RNA-Seq libraries were constructed using the NEB Ultra II RNA Library Prep kit. DNA and RNA sequencing was performed by the Scientific Operations core at the WSI on Pacific Biosciences SEQUEL II (HiFi) and Illumina NovaSeq 6000 (RNA-Seq) instruments. Hi-C data were generated from tissue of inSisNigr2 using the Arima2 kit and sequenced on the Illumina NovaSeq 6000 instrument.

### Genome assembly, curation and evaluation

Assembly was carried out with Hifiasm ( Cheng *et al.*, 2021) and haplotypic duplication was identified and removed with purge_dups ( Guan *et al.*, 2020). The assembly was then scaffolded with Hi-C data ( Rao *et al.*, 2014) using YaHS ( Zhou *et al.*, 2023). The assembly was checked for contamination and corrected using the TreeVal pipeline ( Pointon *et al.*, 2022). Manual curation was performed using using JBrowse2 ( Diesh *et al.*, 2023), HiGlass ( Kerpedjiev *et al.*, 2018) and Pretext ( Harry, 2022). The mitochondrial genome was assembled using MitoHiFi ( Uliano-Silva *et al.*, 2023), which runs MitoFinder ( Allio *et al.*, 2020) or MITOS ( Bernt *et al.*, 2013) and uses these annotations to select the final mitochondrial contig and to ensure the general quality of the sequence.

A Hi-C map for the final assembly was produced using bwa-mem2 ( Vasimuddin *et al.*, 2019) in the Cooler file format ( Abdennur & Mirny, 2020). To assess the assembly metrics, the *k*-mer completeness and QV consensus quality values were calculated in Merqury ( Rhie *et al.*, 2020). This work was done using Nextflow ( Di Tommaso *et al.*, 2017) DSL2 pipelines “sanger-tol/readmapping” ( Surana *et al.*, 2023a) and “sanger-tol/genomenote” ( Surana *et al.*, 2023b). The genome was analysed within the BlobToolKit environment ( Challis *et al.*, 2020) and BUSCO scores ( Manni *et al.*, 2021; Simão *et al.*, 2015) were calculated.

Table 3 contains a list of relevant software tool versions and sources.

**Table 3.** Software tools: versions and sources.

Software tool	Version	Source
BlobToolKit	4.2.1	https://github.com/blobtoolkit/blobtoolkit
BUSCO	5.3.2	https://gitlab.com/ezlab/busco
Hifiasm	0.16.1-r375	https://github.com/chhylp123/hifiasm
HiGlass	1.11.6	https://github.com/higlass/higlass
Merqury	MerquryFK	https://github.com/thegenemyers/MERQURY.FK
MitoHiFi	3	https://github.com/marcelauliano/MitoHiFi
PretextView	0.2	https://github.com/wtsi-hpag/PretextView
purge_dups	1.2.5	https://github.com/dfguan/purge_dups
sanger-tol/genomenote	v1.0	https://github.com/sanger-tol/genomenote
sanger-tol/readmapping	1.1.0	https://github.com/sanger-tol/readmapping/tree/1.1.0
TreeVal	-	https://github.com/sanger-tol/treeval
YaHS	1.2a.1	https://github.com/c-zhou/yahs

### Wellcome Sanger Institute – Legal and Governance

The materials that have contributed to this genome note have been supplied by a Darwin Tree of Life Partner. The submission of materials by a Darwin Tree of Life Partner is subject to the **‘Darwin Tree of Life Project Sampling Code of Practice’**, which can be found in full on the Darwin Tree of Life website here. By agreeing with and signing up to the Sampling Code of Practice, the Darwin Tree of Life Partner agrees they will meet the legal and ethical requirements and standards set out within this document in respect of all samples acquired for, and supplied to, the Darwin Tree of Life Project.

Further, the Wellcome Sanger Institute employs a process whereby due diligence is carried out proportionate to the nature of the materials themselves, and the circumstances under which they have been/are to be collected and provided for use. The purpose of this is to address and mitigate any potential legal and/or ethical implications of receipt and use of the materials as part of the research project, and to ensure that in doing so we align with best practice wherever possible. The overarching areas of consideration are:

• Ethical review of provenance and sourcing of the material

• Legality of collection, transfer and use (national and international)

Each transfer of samples is further undertaken according to a Research Collaboration Agreement or Material Transfer Agreement entered into by the Darwin Tree of Life Partner, Genome Research Limited (operating as the Wellcome Sanger Institute), and in some circumstances other Darwin Tree of Life collaborators.

## Data Availability

European Nucleotide Archive: *Sisyra nigra* (spongefly). Accession number PRJEB62618; https://identifiers.org/ena.embl/PRJEB62618 ( Wellcome Sanger Institute, 2023). The genome sequence is released openly for reuse. The *Sisyra nigra* genome sequencing initiative is part of the Darwin Tree of Life (DToL) project. All raw sequence data and the assembly have been deposited in INSDC databases. The genome will be annotated using available RNA-Seq data and presented through the Ensembl pipeline at the European Bioinformatics Institute. Raw data and assembly accession identifiers are reported in Table 1.

## Author information

Members of the Natural History Museum Genome Acquisition Lab are listed here: https://doi.org/10.5281/zenodo.7139035.

Members of the University of Oxford and Wytham Woods Genome Acquisition Lab are listed here: https://doi.org/10.5281/zenodo.7125292.

Members of the Darwin Tree of Life Barcoding collective are listed here: https://doi.org/10.5281/zenodo.4893703.

Members of the Wellcome Sanger Institute Tree of Life programme are listed here: https://doi.org/10.5281/zenodo.4783585.

Members of Wellcome Sanger Institute Scientific Operations: DNA Pipelines collective are listed here: https://doi.org/10.5281/zenodo.4790455.

Members of the Tree of Life Core Informatics collective are listed here: https://doi.org/10.5281/zenodo.5013541.

Members of the Darwin Tree of Life Consortium are listed here: https://doi.org/10.5281/zenodo.4783558.
