# Supplementary material for: The genome sequence of the Black Spongefly, Sisyra nigra (Retzius, 1783)
Source: Wellcome Open Res. 2023 Nov 9;8:511. [Version 1] doi: 10.12688/wellcomeopenres.20295.1 (PMC11162525; doi:10.12688/wellcomeopenres.20295.1)
Supplement: Peer review history

# Reviewer report: Reviewer response for version 1

**DOI:** 10.21956/wellcomeopenres.22477.r84402

**Published:** 2024-06-08

**Authors:** Xiaodong Cai

**Affiliations:** 1 China Agricultural University, Beijing, China

This article reports the assembly of the chromosome-level genome of *Sisyra nigra*  through sequencing of a  female specimen, with a genome size of 372.6 Mb and a Contig N50 of 3.1 Mb. Additionally, the mitochondrial genome of *Sisyra nigra* was also assembled. All data presented in this study have been released and are available for public access, and detailed methods have been provided. But, I still have some comments on this article.

**Abstract**

 The completeness of the mitochondrial genome, whether it forms a complete circle, is a crucial aspect that should be clarified.  Meanwhile, the abstract should include key assessment metrics for the genome, such as the Contig N50 size and BUSCO completeness.

**Genome sequence report**

 1. The chromosome genome obtained in the article suggests conducting a synteny analysis with the closely related species , to better demonstrate to readers the size and structural features of the chromosomes. Especially regarding sex chromosomes.

 2. The manuscript mentions the assembly of the mitochondrial genome, yet lacks elaboration on its size, structure, and whether it forms a complete circle. It is suggested to provide a brief overview of these aspects.

 3.  This article lacks crucial genome annotation information. it is recommended to add this information to provide readers with a more comprehensive understanding of the genome.

Are sufficient details of methods and materials provided to allow replication by others?

Yes

Is the rationale for creating the dataset(s) clearly described?

Yes

Are the datasets clearly presented in a useable and accessible format?

Yes

Are the protocols appropriate and is the work technically sound?

No

Reviewer Expertise:

Systematic Entomology

I confirm that I have read this submission and believe that I have an appropriate level of expertise to confirm that it is of an acceptable scientific standard, however I have significant reservations, as outlined above.

# Reviewer report: Reviewer response for version 1

**DOI:** 10.21956/wellcomeopenres.22477.r84398

**Published:** 2024-05-25

**Authors:** Luciano Cosme

**Affiliations:** 1 Department of Ecology and Evolutionary Biology, Yale University, New Haven, USA

Review of Scientific Article on the Genome Sequence of the Black Spongefly, Sisyra nigra

 The article "The genome sequence of the Black Spongefly, Sisyra nigra (Retzius, 1783)" presents a comprehensive genome assembly of an individual female Black Spongefly. This study, led by Gavin R. Broad and his team, reports a genome sequence spanning 372.6 megabases, scaffolded into seven chromosomal pseudomolecules, including the X sex chromosome. To ensure high coverage and accuracy, the researchers utilized advanced sequencing techniques such as Pacific Biosciences single-molecule HiFi long reads and Hi-C data. The assembled mitochondrial genome is 16.34 kilobases in length. The final assembly demonstrates high quality, with a Quality Value (QV) of 60.3 and a BUSCO completeness score of 97.1%.

 Importance and Contribution

 The genome sequencing of Sisyra nigra marks the first complete genome assembly for a species of the family Sisyridae, making a significant contribution to the field of genomics and entomology. This study offers valuable insights into the Neuroptera order's genetic makeup and evolutionary history. The detailed assembly provides a robust foundation for future comparative genomics and biological research, particularly in understanding the unique adaptations of the Black Spongefly to its aquatic environment.

 The availability of this genome sequence opens new avenues for studying the genetic basis of the Black Spongefly’s ecological interactions, developmental biology, and evolutionary adaptations. Moreover, the high-quality data and methodologies presented can serve as a reference for genomic studies in other related species, enhancing our overall understanding of insect genomics. The study's meticulous approach to data acquisition and genome assembly sets a benchmark for future projects, ensuring reproducibility and accuracy.

 Overall, this work not only enriches our knowledge of Sisyra nigra but also contributes significantly to the broader scientific understanding of insect biodiversity and evolution.

 ## Questions

 1. Mitogenome Details: The abstract mentions the mitochondrial genome assembly but needs more detail. Can you provide more information about the completeness and characteristics of the mitogenome in the main text?

   

 2. Distribution Clarification: The paper mentions that Sisyra nigra is found in the Holarctic region and North America. Can you clarify whether it is found in both areas or specify any distinctions in its distribution?

   

 3. Chromosome Data: The article states that most of the genome is scaffolded into seven chromosomal pseudomolecules, including the X chromosome. Are there any unique characteristics or findings related to the X chromosome that should be highlighted?

 4. Genome Completeness: The paper mentions a BUSCO completeness score of 97.1%. Can you explain how this score compares to other insect genome assemblies?

 5. Gene Annotations: Are any noteworthy genes or gene families identified in this assembly related to the spongefly’s unique ecological adaptations?

 ## Suggestions

 1. Abstract Enhancement: Enhance the abstract by including specific results and their implications, such as unique findings about the genome structure or any significant annotations.

 2. Methodology Details: While the methods are well-documented, consider adding a brief overview of each critical process in the main text to improve readability and provide context before diving into detailed protocols.

 3. Figures and Tables: Ensure all figures (e.g., Hi-C contact maps, GC-coverage plots) have clear, descriptive legends. This helps readers understand the data at a glance.

 4. Data Availability: In the "Data availability" section, it might be helpful to include direct links to crucial datasets and resources to facilitate access for other researchers.

 5. Comparative Analysis: Include a section or paragraph comparing the Sisyra nigra genome to those of closely related species or other Neuropterida, discussing evolutionary implications or unique genomic features.

Are sufficient details of methods and materials provided to allow replication by others?

Yes

Is the rationale for creating the dataset(s) clearly described?

Yes

Are the datasets clearly presented in a useable and accessible format?

Yes

Are the protocols appropriate and is the work technically sound?

Yes

Reviewer Expertise:

Genomics, population genetics, GWAS

I confirm that I have read this submission and believe that I have an appropriate level of expertise to confirm that it is of an acceptable scientific standard, however I have significant reservations, as outlined above.

# Reviewer report: Reviewer response for version 1

**DOI:** 10.21956/wellcomeopenres.22477.r69903

**Published:** 2023-12-15

**Authors:** Xiumei Lu

**Affiliations:** 1 Shanghai Academy of Agricultural Sciences, Shanghai, China; 2 University of Bristol, Bristol, England, UK

The experiment combined sophisticated methodologies to ensure a reliable and high-quality assembly of the *Sisyra nigra* genome. It represents the first whole genome assembly of *Sisyra nigra*, offering valuable data for potential biological investigations and comparative genomics analyses. 

 In the abstract, the authors mentioned that the mitogenome was also assembled. So, it is necessary to explicitly state more about the mitogenome, indicating whether it is complete or not.

 The authors introduced the significance of this spongefly species and emphasized the outstanding aquatic adaptation of Sisyridae. However, the authors should recheck the distribution of *Sisyra* *nigra*, as there seems to be some confusion regarding whether it is distributed in North America, the Holarctic region, or elsewhere.

Are sufficient details of methods and materials provided to allow replication by others?

Yes

Is the rationale for creating the dataset(s) clearly described?

Yes

Are the datasets clearly presented in a useable and accessible format?

Yes

Are the protocols appropriate and is the work technically sound?

Yes

Reviewer Expertise:

My research areas are systematics, paleobiology and phylogenomics of the superorder Neuropterida (Insecta: Holometabola) to provide critical evidence for the understanding of its evolutionary history.

I confirm that I have read this submission and believe that I have an appropriate level of expertise to confirm that it is of an acceptable scientific standard.
